# Spatio-Temporal Evolutionary Patterns of the Pieridae Butterflies (Lepidoptera: Papilionoidea) Inferred from Mitogenomic Data

**DOI:** 10.3390/genes14010072

**Published:** 2022-12-26

**Authors:** Fanyu Wei, Wenxiang Huang, Lin Fang, Bo He, Youjie Zhao, Yingming Zhang, Zufei Shu, Chengyong Su, Jiasheng Hao

**Affiliations:** 1 College of Life Sciences, Anhui Normal University, Wuhu 241000, China; 2 Guangdong Chebaling National Nature Reserve Administration Bureau, Shaoguan 512500, China

**Keywords:** Pieridae butterflies, mitochondrial genome, phylogeny, divergence times, historical biogeography

## Abstract

Pieridae is one of the largest and almost cosmopolitan groups of butterflies, which plays an important role in natural ecosystems; however, to date, its phylogeny and evolutionary history have not been fully resolved. In this study, we obtained the complete or nearly complete mitochondrial genomes of 100 pierid taxa (six newly sequenced, sixty extracted from the whole-genome data, and thirty-four directly available from GenBank). At the same time, for the first time, we conducted comparative mitogenomic and phylogenetic analyses based on these mitogenomic data, to further clarify their spatio-temporal evolutionary patterns. Comparative mitogenomic analysis showed that, except for *cox2*, the GC content of each of the 13 protein-coding genes (PCGs) in the rapidly diverging subfamily Pierinae was higher than in its sister group Coliadinae. Moreover, the *dN*/*dS* values of nine genes (*atp6*, *atp8*, *cox1*, *cox3*, *cob*, *nad1*, *nad3*, *nad5*, and *nad6*) in Pierinae were also relatively higher than those in its sister group, Coliadinae. Phylogenetic analysis showed that all the resultant phylogenetic trees were generally in agreement with those of previous studies. The Pierinae family contained six clades in total with the relationship of (Leptosiaini + (((Nepheroniini + Arthocharidini) + Teracolini) + (Pierini + Elodini))). The Pieridae originated in the Palearctic region approximately 72.3 million years ago in the late Cretaceous, and the subfamily Pierinae diverged from this family around 57.9 million years ago in the Oriental region, shortly after the K–Pg mass extinction event; in addition, the spatio-temporal evolutionary patterns of Pierinae were closely correlated with geological events and environmental changes, as well as the host plant coevolutionary scenario in Earth’s history. However, some incongruencies were observed between our results and those of previous studies in terms of shallow phylogenies for a few taxa, and should be further investigated.

## 1. Introduction

As herbivores and pollinators, butterflies play a fundamental role in terrestrial ecosystem and are also indicators of environmental change [1]. Additionally, butterflies serve as models for research on community ecology, speciation, biogeography, and plant-insect interactions. Their diversification are presumed to have coevolved with angiosperms insects [2,3,4]. Pieridae is one of the major butterfly groups, comprising approximately 1100 described species in 85 genera worldwide [5,6,7]. In previous decades, many efforts were made to clarify the phylogeny of Pieridae by using morphological, molecular (mitochondrial and nuclear genes), or combined data, and currently, the monophyly of its four subfamilies (Pseudopontiinae, Dismorphiinae, Coliadinae, and Pierinae) are well established [6,7,8]. However, the relationships among Pieridae crown lineages, especially some tribes or genera within the rapidly diverging subfamily Pierinae, remain relatively unclear. The Pierinae species is divided into two tribes (Anthocharidini and Pierini) primarily based on detailed morphological studies [8,9]. Braby et al. (2006) were the first to examine the systematics of Pierinae in detail using molecular data, and this subfamily was divided into four main lineages (Anthocharidini, Pierini, *Colotis,* and *Leptosia*) with four genera (*Leptosia*, *Elodina*, *Dixeia*, and *Belenois*), which were not convincingly identified [6]. Wahlberg et al. (2014) subsequently revised this classification through relatively comprehensive taxon sampling: Using one mitochondrial and seven nuclear protein-coding genes, they resurrected the tribal status of Teracolini introduced by Reuter [10], established three new tribes (Elodinini, Leptosiaini, and Nepheroniini), and further indicated that the subfamily Pierinae should be divided into six tribes [8]. However, some paraphyletic groups were found in their results, and the basal nodes of Pierinae (*Leptosia*, *Elodina*, and *Nepheronia* + *Pareronia*) were largely unresolved. Considering these problems, together with the fact that some non-monophyletic genera (especially in the Pierini tribe) are under continuous controversy [6,11], it is necessary to obtain more genetic data, broader taxon sampling, and more representative morphological characteristics to clarify these issues [6,7,11,12].

Owing to scarce fossil records [6,7], the molecular dating for the origin and divergence of Pieridae and their subsequent ancestral geographical reconstruction also revealed inconsistencies among previous studies [6,13,14,15]. Recently, de Jong et al. (2017) revised the fossil records for butterfly species, providing a relatively standard framework for different fossil specimens as well as their taxonomic placement. Two Pieridae fossils (*Vanessa pluto* and *Miopieris talboti*) were confirmed with their deposits preserved in Miocene (20.4–16 Mya) [16] and under these criteria, a rough estimation of the time of origin of Pieridae was in the upper Cretaceous, according to Espeland et al. (2018) and Chazot et al. (2019) [17,18]. As for the phylogeographic inference, in a study by Braby et al. (2006), the two closely related subfamilies Pseudopontiinae and Dismorphiinae were estimated to have likely originated in western Gondwana and, owing to their strictly current geographic distribution, likely diverged either to the Afrotropical, Neotropical, or Palearctic regions [6]. However, as indicated by Braby et al., it proved challenging to reach a clear consensus on their strong migratory tendencies and poorly resolved phylogenetic relationships for the other two larger subfamilies, Coliadinae and Pierinae. Nevertheless, the subtribe Aporiina was estimated to likely originate in southern Gondwanan, along with *Delias*, a diverse and widespread clade originating in the Australian region, with at least seven dispersal events across Wallacean to the Oriental region [19,20]. Müller et al. (2012), using more taxon sampling, subsequently indicated that the Australian Plate is a plausible site of origin for the genus *Delias* [21]. More recently, Gash et al. (2020) revealed that the group *Mylothris* originated in east-central Africa, and from there, spread to other parts of Africa [22].

The typical insect mitogenome is a circular and compact molecule, ranging from 14 to 20 kb in size and including thirteen protein-coding genes (PCGs), two ribosomal RNA genes (rRNAs), twenty-two transfer RNA genes (tRNAs), and a large non-coding region (also known as control region) [23,24,25]. Owing to its unique features of relatively small size, maternal inheritance, low recombination rate, and accelerated substitution rate [26,27], it has been extensively used in studies of deep and low-level insect phylogeny, molecular evolution, and other related areas [28,29,30]. In addition, as suggested in a previous study, dense taxon sampling is essential for phylogenomic tree reconstruction since insufficient sampling may lead to wrong clade relationships [31]. However, to date, only 34 complete or nearly complete mitogenomes have been sequenced for Pieridae. Moreover, the most recent mitogenomic phylogenetic study for Pieridae only contains 22 pierid taxa, covering 13 genera [32], which is not sufficient for a full understanding of the Pieridae evolutionary process at the mitogenome level.

In this study, we determined newly discovered, complete mitochondrial genomic sequences of six pierid species, namely *Ixias pyrene*, *Cepora nerissa*, *Cepora nadina*, *Pareronia anais*, *Prioneris thestylis*, and *Dercas lycorias*, to fill the gap in research on the mitochondrial genome of these controversial genera. Through this process, we extracted and annotated the complete mitochondrial genome of 60 pierid species, covering 42 genera, using the whole-genome data available from GenBank. Thus, taken together, a total of 100 mitochondrial genomes, covering 56 genera, were first used to reconstruct the phylogenetic trees of the main lineages of pierid butterfly species; then, their divergence times were estimated with relaxed molecular dating methods using multiple calibrations, and their ancestral geographic distributions were constructed based on current geographic distributions, to further clarify their spatio-temporal evolutionary patterns.

## 2. Materials and Methods

### 2.1. DNA Extraction, Mitogenome Sequencing, and Assembly

The adult individuals of six newly identified pierid butterfly species (Supplementary Table S1) were collected and preserved in 100% ethanol for fixation and stored at −20 °C (College of Life Sciences, Anhui Normal University) until subsequent experiments. The total genomic DNA was extracted from a single specimen with a QIAGEN DNeasy Blood and Tissue Kit (Hilden, Germany), following the manufacturer’s protocols. Briefly, a total amount of 0.2 μg DNA per sample was used as input material for DNA library preparations, and the sequencing library was generated using NEB Next^®^ Ultra™ DNA Library Prep Kit for Illumina (NEB, San Diego, CA, USA) following the manufacturer’s protocols. The genomic DNA sample was fragmented via sonication to an average size of 350 bp and was sequenced on an Illumina platform (Illumina novaseq6000; Novegene, Tianjing, China) with 150 bp generated paired-end reads. Each sample was generated about 10 Gb of raw data, together with the 60 whole-genome data directly downloaded from GenBank, which were filtered out low quality reads, adapter contamination and ambiguous bases using fastp v0.20.0 [33] with the parameters of “−q 15 −n 10 −u 40”. Subsequently, a total of 66 mitogenomes were assembled using the GetOrganelle v1.7.0 [34] program with default settings (minimum and maximum k values of 21 bp and 155 bp).

### 2.2. Gene Annotation and Mitogenome Analysis

The secondary structures of tRNAs were predicted using MITOS2 [35] with the invertebrate mitochondrial genetic code. The PCGs, rRNAs, and control region boundaries were determined by the positions of tRNAs. In addition, to ensure more accurate gene boundaries, PCGs and rRNA genes were verified through sequence comparison with other Pieridae mitogenomes and then confirmed with manual calibration using SeqMan [36]. PCGs were also translated into amino acids based on the invertebrate mitochondrial genetic code. The tandem repeats of the control region were predicted using the Tandem Repeats Finder online server (http://tandem.bu.edu/trf/trf.html, accessed on 18 March 2022) [37]. The base composition and relative synonymous codon usage (RSCU) of the 100 mitogenomes and the GC content of each gene for each species were analyzed using DAMBE 7 [38]. The ratio of non-synonymous (*dN*) to synonymous substitutions (*dS*) for each PCG among species was calculated with DnaSP v6.0 [39].

### 2.3. Measures of Nucleotide Variation

The substitution saturation of the 13_PCGs (13 protein-coding genes), 15_genes (the combination of 13 PCGs and 2 rRNAs), and 13PCGs_codon3 (the third position of 13 PCGs) were also tested in DAMBE 7 [38], with the GTR model selected as a reference model. Moreover, mitogenomes have proven to be powerful in defining the major lineages at the subfamily level and below, while they are usually affected by shifts in both evolutionary rates and nucleotide composition. In addition, owing to the shifts that existed in both the evolutionary rate and nucleotide composition, the heterogeneity of sequence divergence within datasets relative to an external reference (outgroup) was analyzed with AliGROOVE v1.07 [40]. The default sliding window size was used, with indels in the nucleotide dataset treated as ambiguity, to investigate the heterogeneity in these mitogenomic data.

### 2.4. Phylogenetic Analysis

Phylogenetic analysis was conducted with two datasets: (1) 13_PCGs and (2) 15_genes. In our analysis, 100 species representing three subfamilies, nine tribes of Pieridae, and fifty-six genera were used to reconstruct the phylogenetic trees, with three Papilionidae and three Hesperiidae species used as the outgroups (Supplementary Table S1). Nucleotide sequences for each of the 13 PCGs were translated into amino acids, separately aligned with muscle implemented within MEGA 7 [41]; the rRNAs were aligned using MAFFT 7.490 [42], with the L-INS-i algorithm. All the unreliably aligned sequences were eliminated using Gblocks 0.91b [43], with the aligned data of PCGs and rRNAs from each locus concatenated with SequenceMatrix v1.9 [44].

The maximum likelihood (ML) and Bayesian inference (BI) methods were used to reconstruct the phylogenetic tree. ML analysis was performed using IQ-TREE with 10,000 bootstrap replicates [45], and the best-fit partitioning schemes and substitution models (Supplementary Table S2) were recommended using ModelFinder [46], implemented in the IQ-TREE program. The support for each node of the tree (BPs) was evaluated via the bootstrap test with 1000 iterations. BI analysis was conducted in MrBayes v3.2.7 [47], and the nucleotide substitution models and best-fit partitioning schemes (Supplementary Table S2) were simultaneously recommended by PartitionFinder v2.1.1 [48], using the “greed” algorithm. Using the Akaike information criterion (AICc), two parallel analyses with four independent Markov chains (three hot chains and one cold chain) were conducted. The samples included 2 million generations, with sampling performed for every 100 generations when the convergence of MCMC chains was achieved (i.e., the average standard deviation of split frequencies was less than 0.01); the first 25% of the sampled trees were discarded as “burn-in” samples. The confidence values of the tree nodes were estimated as the posterior probabilities (PPs). All the generated trees were displayed using FigTree v1.4.4 (http://tree.bio.ed.ac.uk/software/figtree/, accessed on 24 June 2022) software.

### 2.5. Divergence Time Estimation

In the present study, two fossil calibrations (*V. pluto* and *M. talboti*) were used to estimate the times of divergence, which were recently critically reviewed by de Jong [16]. Apomorphic character or character combinations diagnostic of extant clades were observed, thereby allowing reliable fossil allocations on phylogenetic trees to provide minimum ages to the corresponding nodes. Their geological times dated back 20.4 to 16.0 million years ago, respectively, both with a log-normal prior. In addition, two secondary calibration points of relevant host plants (Fabaceae and Brassicaceae) [49] were used in this study as the maximum constraint, owing to their coevolutionary scenarios [2,50,51].

The divergence times were identified using BEAST v1.10.4 [52] with the GTR+I+G model. The analysis assumed a birth–death speciation process and used an uncorrelated lognormal relaxed clock. Markov chain Monte Carlo (MCMC) chains were run for 30 million generations, with the parameters sampled every 3000 generations. Tracer v1.7.1 [53] was used to assess the convergence of the chains and effective sample size for each parameter, which was ≥ 200 for all the samples. The first 25% of the trees were discarded as “burn-in”, and the node ages with upper and lower 95% posterior density (HPD) were calculated using TreeAnnotator v1.10.4. In addition, a lineage-through-time (LTT) analysis [54] was performed to determine the tempo of the diversification of the species and to assess its possible relation to temperature changes and geological events in Earth’s history. The LTT plot of the lineage numbers against the divergence time was constructed using the ape and ggplot2 packages in R v4.1.2 [55].

### 2.6. Ancestral Area Reconstruction

The biogeographic realms in this study generally contained six areas, as previously demonstrated [56]: Nearctic (A), Palearctic (B), Afrotropic (C), Oriental (D), Australasia (E), and Neotropical (F). We compiled the distributional data for the sampled taxa according to their modern distributional areas and constrained the common ancestral areas, which were previously studied [19,21,22]. We performed the Bayesian binary MCMC (BBM) method of biogeographic ancestral area reconstruction implemented in RASP v4.x [57], using a guiding tree inferred from BEAST and the outgroup taxa excluded. The BBM analysis was run for 5 million cycles, using 10 chains, and sampling every 100 cycles. The temperature was set at 0.1, with a fixed JC model. The maximum number of areas for all the nodes was set to three, and the time–event curve was calculated by the “Time-Calculate” option in RASP. The data for each node were plotted in pie charts.

## 3. Results and Discussionteen 

### 3.1. Characterization of Mitogenomes

**Figure 1:**
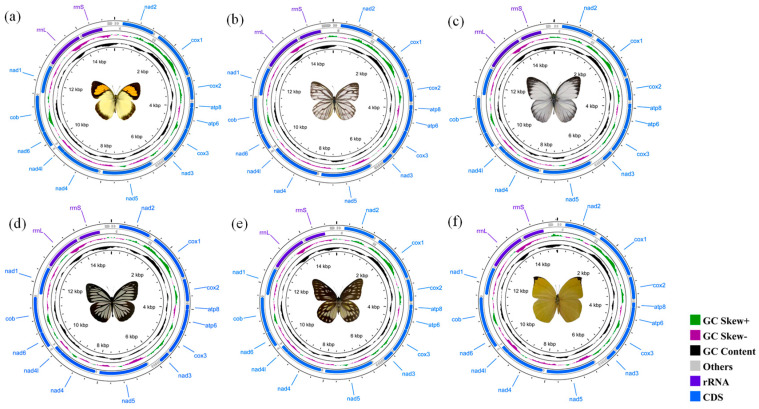
The circle maps of the complete mitochondrial genomes of (**a**) *I. pyrene*, 15,178 bp, (**b**) *C. nerissa,* 15,185 bp, (**c**) *C. nadina*, 15,216 bp, (**d**) *P. anais,* 15,161 bp, (**e**) *Pr. thestylis*, 15,174 bp, and (**f**) *D. lycorias,* 15,242 bp. In total, thirteen PCGs and two rRNAs were considered; the arrow indicates the direction of the gene from start to end.

The six complete mitogenome sequences of *I. pyrene* (15,178 bp), *C. nerissa* (15,185 bp), *C. nadina* (15,216 bp), *P. anais* (15,161 bp), *Pr. thestylis* (15,174 bp), and *D. lycorias* (15,242 bp) were typical of insect mitogenomes, with their sizes falling within the range of other identified butterflies (Figure 1). They all contain thirteen PCGs, two rRNAs, twenty-two tRNAs, and a non-coding region, with the gene order and arrangement identical to the presumed ancestral insect mitogenomes. The resulting assembly and annotations were deposited in GenBank, with accession numbers OP779721 (*C. nerissa*), OP779722 (*C. nadina*), OP779723 (*P. anais*), OP779724 (*Pr. thestylis*), OP779725 (*D. lycorias*), and OP779726 (*I. pyrene*), respectively.

**Figure 2:**
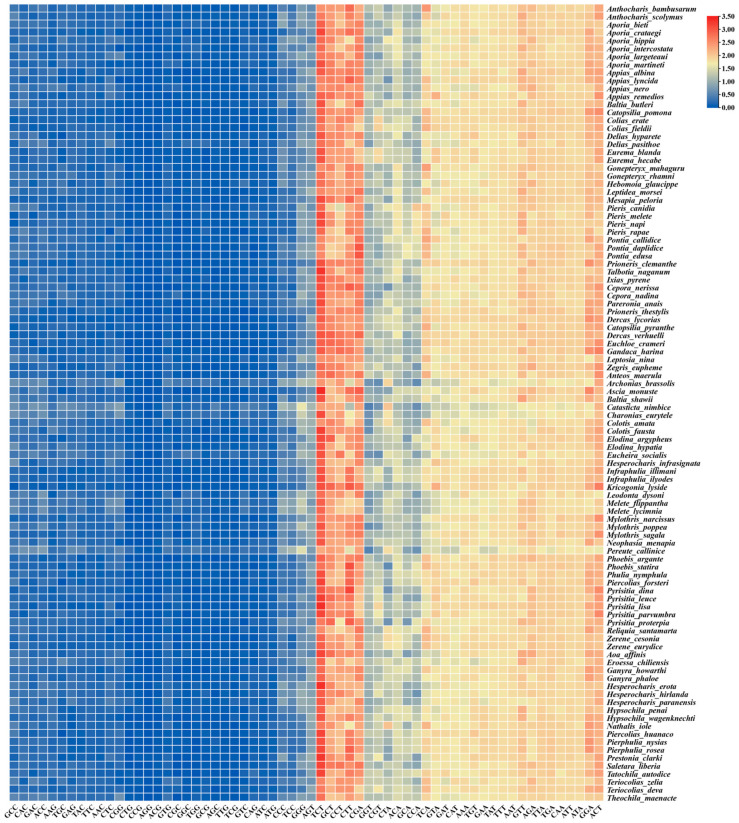
The relative synonymous codon usage (RSCU) of 13 PCGs in the mitogenomes of Pieridae. The *x*- and *y*-axis represent the codon type and species name, respectively. The legend in the upper-right corner represents the usage frequency of synonymous codons.

**Figure 3:**
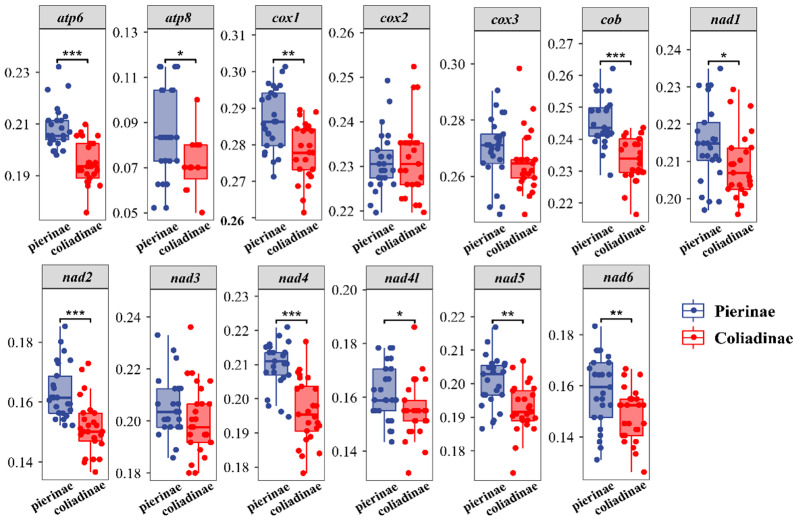
Wilcoxon rank-sum test comparison of GC contents of 13 mitochondrial protein-coding genes between the subfamilies Coliadinae and Pierinae (* denotes *p* ≤ 0.05; ** denotes *p* ≤ 0.01; *** denotes *p* ≤ 0.001).

**Figure 4:**
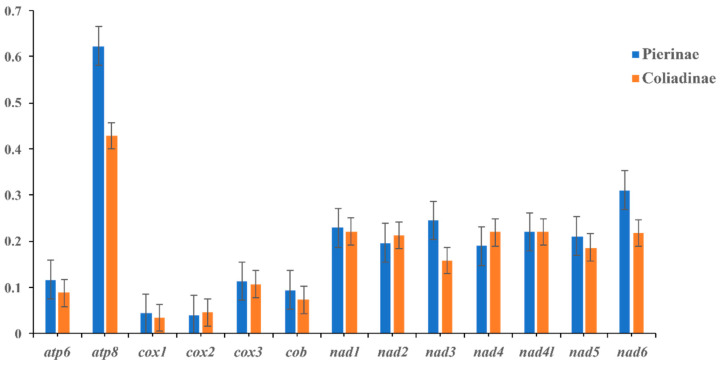
Evolutionary rate of each PCG among Pierinae and Coliadinae species. The ratio of the rate of non-synonymous substitutions to the rate of synonymous substitutions (*dN*/*dS*) for each PCG.

The RSCU analysis of the PCGs showed that the third codon position among the five most commonly used codons (TCT, GCT, CCT, CTT, and CGA) of the whole Pieridae group is A or T (Figure 2), indicating a strongly positive AT skew. Notably, the GC content of each of the 13 PCGs (except the *cox2* gene) was higher in Pierinae than in Coliadinae (Figure 3), and the GC contents of the four PCGs (*atp6*, *cob*, *nad2*, and *nad4*) in the subfamily Pierinae were significantly higher than those in Coliadinae (*p* < 0.01, Wilcoxon rank-sum test). The ratio of non-synonymous (*dN*) to synonymous substitutions (*dS*) for the PCGs was assessed separately for Coliadinae and Pierinae. Furthermore, the average *dN*/*dS* value of nine genes (atp6, *atp8*, *cox1*, *cox3*, *cob*, *nad1*, *nad3*, *nad5*, and *nad6*) in Pierinae was higher than in Coliadinae (Figure 4). We believe that the elevated GC content might be correlated with a high mutation rate, which triggered the rapid development of the subfamily Pierinae and differentiated it from the Pieridae.

### 3.2. Assessment of Sequence Variation

**Figure 5:**
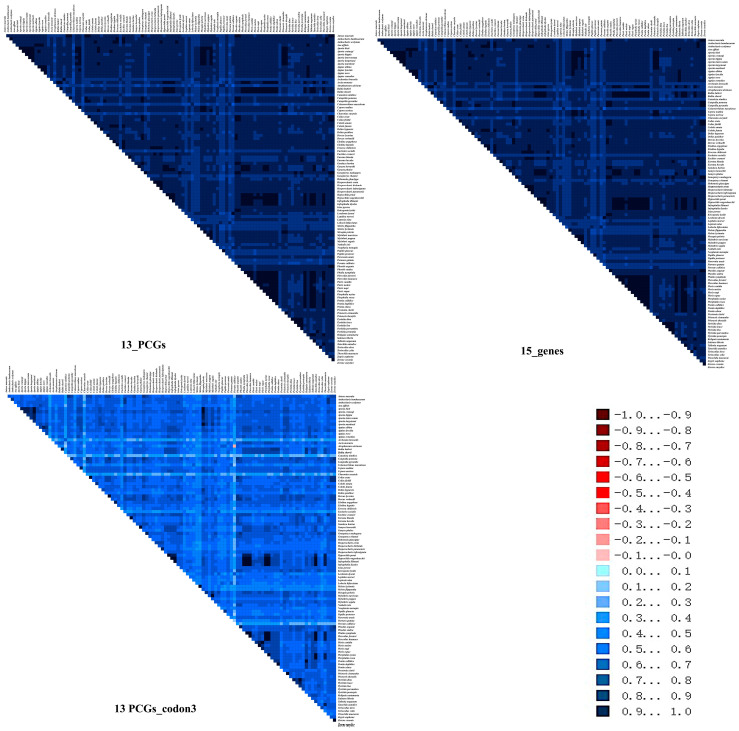
Heterogeneous sequence divergence of mitochondrial genomes for different datasets. The mean similarity score between sequences is represented by a colored square. The scores range from −1, indicating full random similarity, to +1, indicating non-random similarity, which is visualized by a color range from dark red (−1) to dark blue (+1).

The plots of uncorrected pairwise sequence divergence against the divergence calculated using a GTR model (Supplementary Figure S1) showed that, even though 13PCGs_codon3 diverged faster for the transversions than for transitions, because of the high level of mutational saturation, they were not saturated, indicating that 13_PCGs and 15_genes are suitable for the direct analysis of deep divergences in the Pieridae phylogeny. The heterogeneity of sequence variation was assessed with AliGROOVE v1.07 [40], separately for different datasets. All the datasets yielded extremely low scores (Figure 5). In general, the mitogenomes had relatively low heterogeneity of sequence composition for most pairwise comparisons between the sequences for the 13_PCGs, 15_genes, and 13PCGs_codon3. This was observed through a comparison of the pairwise sequence divergence of individual terminals to terminals outside of the focal group against the same measure of divergence over the entire data matrix.

### 3.3. Phylogenetic Relationships

**Figure 6:**
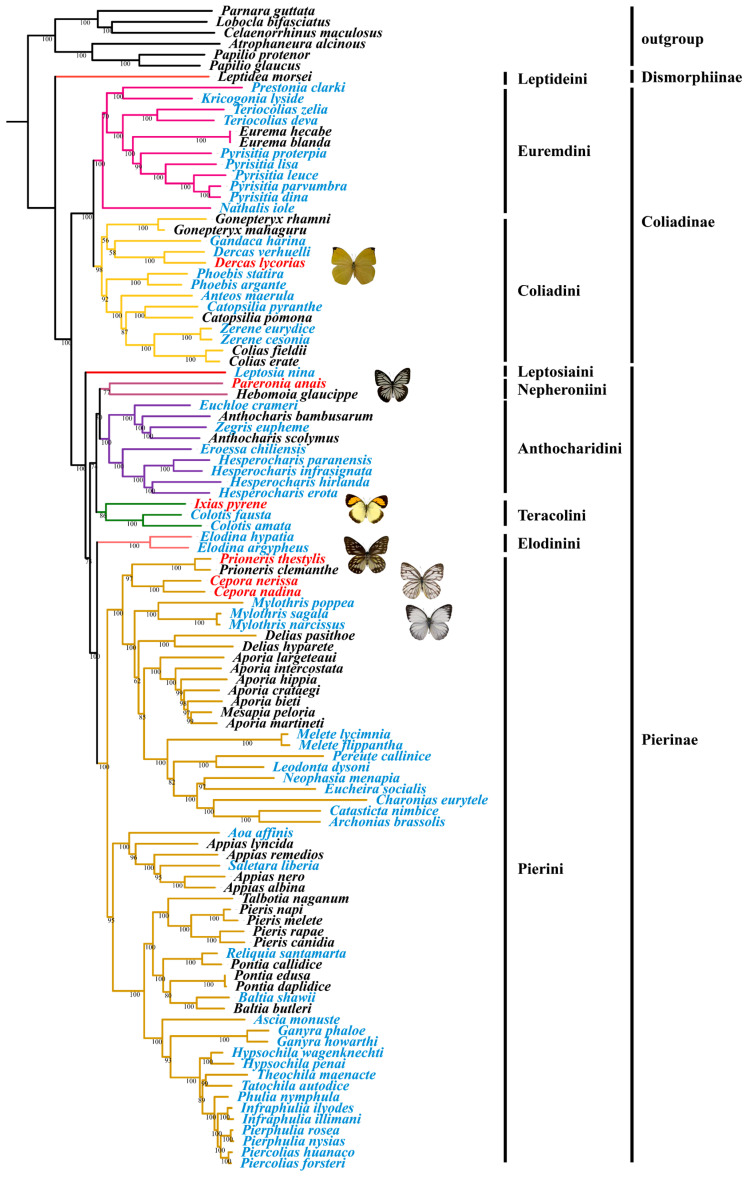
Phylogenetic relationships of the 100 Pieridae species from maximum likelihood (ML) analysis based on 13 mitochondrial protein-coding genes; bootstrap values are indicated at each node. The species sequenced and extracted from the whole genome are marked in red and blue, respectively.

In the present study, we determined the phylogenetic trees of Pieridae based on 100 mitochondrial genomes (covering 56 genera), with both datasets (13_PCGs and 15_genes) across different methods (ML and BI phylogenetic analyses). This analysis yielded identical tree topology, with strong evidence for most of the nodes (BS = 80–100, PP = 0.95–1.00). Our results corroborated the phylogenetic data of the Pieridae butterfly family found in previous studies [6,7,11,12,32]; furthermore, the phylogenetic relationships of more taxa groups were revealed. Specifically, our results indicated that three subfamilies, namely Dismorphiinae, Coliadinae, and Pierinae, were strongly supported as monophyletic groups (BS = 100, PP = 1.00), with Dismorphiinae recovered as a sister group with Coliadinae + Pierinae, and Coliadinae recovered as a sister group with Pierinae (Figure 6).

In this study, within the subfamily Coliadinae, two monophyletic tribes (Euremdini and Coliadini) covering a total of 14 genera were well supported. The tribe Euremdini was revealed to have six genera, with the relationship of (*Nathalis* + ((*Kricogonia* + *Prestonia*) + ((*Eurema* + *Pyrisitia*) + *Teriocolias*))), while the tribe Coliadini comprised eight genera, with the relationship of ((*Gonepteryx* + (*Gandaca* + *Dercas*)) + (*Phoebis* + (*Anteos* + (*Catopsilia* + (*Colias* + *Zerene*))))), which was generally consistent with the results by Braby et al. [6] and Wahlberg et al. [7], except for the genus *Dercas*, which was a sister genus to *Gandaca*, and the clade *Gandaca* + *Dercas*, which was a sister clade to *Gonepteryx* [6,7]. In another study, the genus *Kricogonia* was considered more closely related to *Phoebis* in terms of its morphological features [9]. However, our study supports the findings of the previous studies based on molecular sequence data indicating that *Kricogonia* and *Phoebis* were nested within the tribes Euremdini and Coliadini, respectively [6,7].

The internal phylogeny of the subfamily Pierinae was largely unresolved in previous studies, probably due to their rapid radiation [6,7,11,58]. For example, the phylogenetic positions for some of its taxa (*Leptosia*, *Elodina,* and *Nepheronia* + *Pareronia*) remained unclear [7]. This subfamily is commonly divided into two tribes (i.e., Anthocharidini and Pierini) primarily based on detailed morphological characteristics [8,9], while Braby et al. (2006) proposed that they should be divided into four main lineages (i.e., Anthocharidini, Pierini, *Colotis*, and *Leptosia*) [6]. Wahlberg et al. (2014) subsequently divided the subfamily into six tribes (i.e., Elodinini, Leposiaini, Nepheroniini, Teracolini, Anthocharidini, and Pierini) based on one mitochondrial and seven nuclear gene sequence data [7], while their relationships were far from clear [11,17]. Our study, based on relatively more comprehensive taxon sampling and mitogenomic sequences, showed that the six tribes of Pierinae were recovered with the relationships of (Leptosiaini + (((Nepheroniini + Anthocharidini) + Teracolini) + (Pierini + Elodinini))). This implied that the Nepheroniini was sister to Anthocharidini, which was supported by their apomorphies, such as palpus with a short, oval third joint; forewing with vein R1 arising from the discal cell branches of R3, R4, and R5 stalked; hindwing with long humeral vein; penis lightly bent without basal prong, etc. [7,9]. Pierini was identified as a sister tribe to Elodinini, which was also supported by their shared morphological characteristics, e.g., the third joint of palpus being longer than the second, often abrupt antennal club, often long humeral vein, low discocellular (*ldc*) longer than middle discocellular (*mdc*), etc. [7,9].

Our study showed that the tribe Anthocharidini contained two monophyletic clades: one clade comprised the genera *Euchloe*, *Anthocharis*, and *Zegris*, and the other comprised *Eroessa* and *Hesperocharis*. However, the genus *Anthocharis* was identified as a non-monophyletic group in this study since strong evidence indicated that *Zggris eupheme* was nested within this genus, implying that the genus *Zggris* should be synonymized with *Anthocharis*. Additionally, our study showed that the genus *Hebomoia* was clustered with *Pareronia* in the tribe Nepheroniini rather than in Anthocharidini, thus supporting the results of Braby et al. [6] but refuting the suggestion of its Anthocharidini grouping [6,7,11,12,32]. The tribe Pierini was found to contain three subtribes with their relationship of (Appiadina + (Aporiina + Pierina)), in agreement with the results of previous studies [6,7]. For the internal phylogeny of the subtribe Aporiina, the grouping of (*Prioneris* + *Cepora*) was sister to the remaining taxa, in agreement with previous studies [6,7]. Moreover, both *Aporia* and *Appias* were identified as paraphyletic groups. Since the monotypic *Mesapia* is nested within *Aporia*, our results supported that *Mesapia* should be synonymized with *Aporia* [8,9,11,12,13,32,59]. Likewise, *Saletara liberia* was also found to be nested within the genus *Appias*, which was consistent with previous studies [6,9,60], except for the study by Wahlberg et al. [7]. Thus, *Saletara* should be classified under *Appias* or be synonymized with *Appias*. However, more studies with taxon sampling and the inclusion of both molecular and morphological data are needed to test these hypotheses.

### 3.4. Divergence Time Estimates

**Figure 7:**
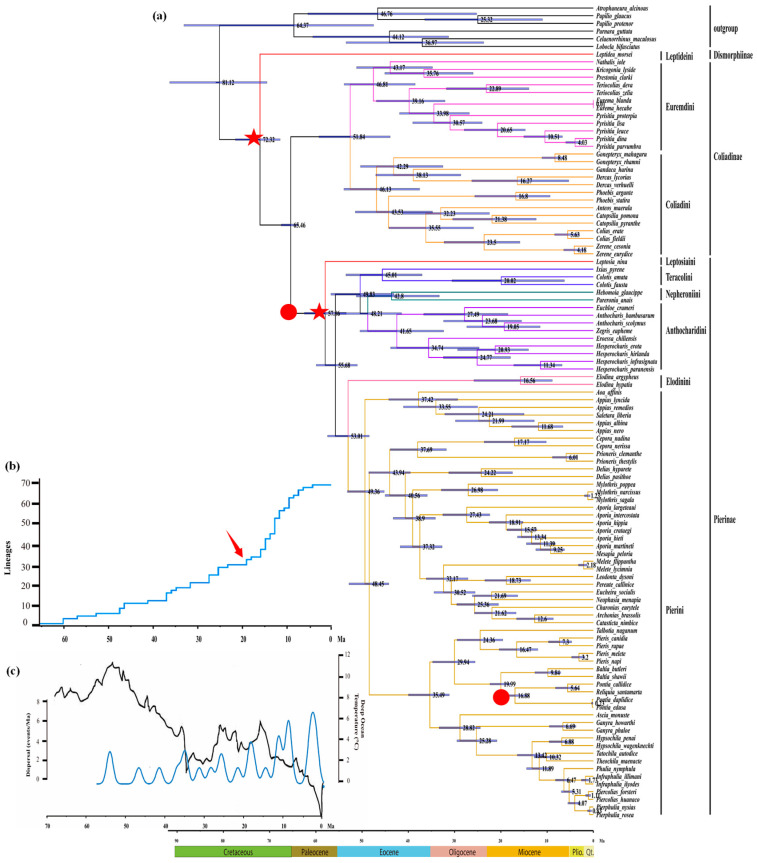
Estimated divergence dates and diversification rate of Pieridae and their association with geological and climatic events: (**a**) estimated time tree of the Pieridae; the blue bars represent 95% posterior density intervals for the node age, and placement of two fossil calibrations and two secondary calibrations are indicated by red circles and red stars, respectively); (**b**) the LTT plot of Pierinae diversification, and the point of a sharp increase are marked with a red arrow; (**c**) climatic–dispersal curve for Pierinae. Deep ocean temperatures (black curve, as a proxy for global temperature) were derived from oxygen isotopes corrected for variation in global ice volume; the time–event curve (blue curve) shows the changes in the frequency of dispersal.

The estimations of Pieridae divergence times significantly varied among the previous studies [6,13,17,18] due to the unsuitable fossil calibrations adopted and thus poorly inferred phylogenies [61,62]. Our study showed that the most recent common ancestor (MRCA) of Pieridae originated at about 72.3 Mya (95% CI: 67.7 to 77.4 Mya) during the Upper Cretaceous (Figure 7a), generally congruent with the latest estimate by Chazot et al. [18]. The split between Coliadinae and Pierinae occurred at about 65.5 Mya (95% CI: 63.7 to 67.5 Mya) during the Cretaceous–Paleogene (K–Pg) boundary, and these subfamilies began to diversify at about 51.8 Mya (95%CI: 43.9 to 59.3 Mya) and 57.9 Mya (95% CI: 53.4 to 62.5 Mya), respectively. Thus, the Pieridae ancestral lineages might have survived by small populations in geographically restricted areas at the K–Pg boundary, with a relatively long delay before they began to diversify [14,56]. This finding supports the view that the K–Pg event had a huge impact on phytophagous insects, including some groups of butterflies [63], but refutes the suggestion that the Pierinae diverged shortly after the appearance of the Brassicales (90–85 Mya) [6,64]. This case of asynchrony between the evolutionary history of animal taxa and plant migrations (at least 20 Ma earlier) was also revealed in a previous study [65].

The tribe-level lineages within Pierinae and Coliadinae were estimated to have diverged mostly during the late Paleocene and early Eocene periods (Figure 7a). Within the subfamily Coliadinae, the tribes Euremdini and Coliadini began to diverge at about 46.8 Mya (95% CI: 38.5 to 53.9 Mya) and 46.1 Mya (95% CI: 43.9 to 59.3 Mya), respectively; in comparison, within the subfamily Pierinae, the divergence between Leptosiaini and the remaining lineages of Pierinae occurred at about 57.9 Mya, and the divergence between the grouping of (Nepheroniini + Anthocharidini) and Teracolini occurred at about 49.8 Mya (95% CI: 43.1 to 56.8 Mya). Furthermore, the split between the tribe Nepheroniini and Anthocharidini occurred at about 48.2 Mya (95% CI: 41.5 to 55.3 Mya), and the split between the Pierini and Elodinini occurred at about 49.4 Mya (95% CI: 45.2 to 53.9 Mya).

The empirical LTT plots (Figure 7b) of the entire Pierinae clade revealed a relatively slow but constant increase from 65 to 20 Mya, followed by a sharp increase in lineage diversification during the early Miocene at about 20 Mya. This might have resulted from the nearly simultaneous onset of diversification for the largest tribe Pierini. The significantly increased dispersal events shown by the time–event curves (Figure 7c) were probably correlated with the fluctuations and gradual cooling of the average global temperature [66], as well as the host plant (Brassicales) dispersals [67] during this period.

### 3.5. Ancestral Area Reconstruction

**Figure 8:**
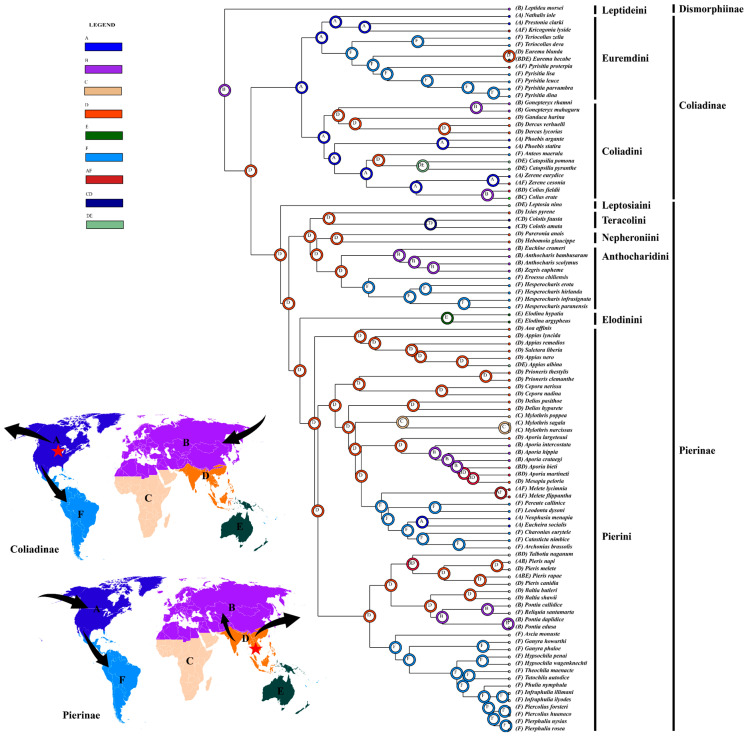
Results of the ancestral area reconstruction of Pieridae from BBM analysis. Biogeographical realms: Nearctic (A), Palearctic (B), Afrotropic (C), Oriental (D), Australasia (E), and Neotropical (F). The originating areas of Coliadinae and Pierinae are indicated by red stars.

Pierid butterflies are distributed worldwide but are unevenly distributed throughout the major zoogeographical regions. Our study showed that under the improved phylogenetic framework using the BBM method, the MRCA of the family Pieridae was likely to have originated in the Palearctic region during the Campanian period in the late Cretaceous, and the early evolution of its lineages occurred in this continent until the end of the Cretaceous and then dispersed into other ecoregions (Figure 8).

In terms of the higher taxa recognized in the family, the four subfamilies Dismorphiinae, Pseudopontiinae, Coliadinae, and Pierinae might have markedly different evolutionary patterns among zoogeographical regions. As a previous study has shown, Dismorphiinae probably originated in the western Gondwana region around 70 million years ago, during which South America and Africa were still connected [6]. Later, they dispersed mainly into the Neotropical region, with a few lineages into the Palaearctic. As for the Pseudopontiinae, previous studies showed that they are closely related to Dismorphiinae or Pierinae [6,7,17]. Nevertheless, they are currently endemic to Africa [6]; thus, their originating area is still a mystery. However, the originating areas of the two other large subfamilies, Coliadinae and Pierinae, due to their strong migratory tendencies and insufficient taxon sampling in previous studies, are also far from clear. The MRCA of Coliadinae and Pierinae was found to have dispersed from the Palearctic into the Oriental region through a land bridge that connected to the Oriental region resulting from the India–Eurasia collision after the late Cretaceous [68,69]. The MRCA of Coliadinae then dispersed from the Oriental region into the Nearctic region between 65.5 and 51.8 Mya, most likely via the Bering Land Bridge (BLB) [70]. This dispersal into new areas may have prevented them from K–Pg global extinction by increasing the cumulative chance of lineage survival. In Coliadinae, the most likely ancestral region for the tribe Euremdini was found to be the Nearctic region, and among this tribe, one clade (*Nathalis* + (*Kricogonia* + *Prestonia*)) was endemic to the Nearctic region, while the other clade (*Teriocolias* + (*Pyristia* + *Eurema*)) dispersed into the Neotropical region through the Isthmus of Panama (IP) at about 39 Mya in late Eocene. This coincided with a floral shift in the Neotropical region due to the more temperate and suitable habitats of tropical biomes [71]. As for the other tribe Coliadini, their most likely ancestral region was also the Nearctic, and one of its clades (*Phoebis* + (*Anteos* + (*Catopsilia* + (*Colias* + *Zerene*)))) was endemic to the Nearctic region, whereas the other clade ((*Gonepteryx* + (*Gandaca* + *Dercas*)) dispersed backed to the Oriental region at about 42.3 Mya.

The MRCA of Pierinae diverged in the Oriental region after the K–Pg mass extinction around 65.5 million years ago, and their ancestral lineages may have stayed and survived by small populations with a relatively long delay before they began to diversify. Our results showed that the most likely ancestral region for ((Nepheroniini + Anthocharidini) + Teracolini)) and (Pierini + Elodinini) was the Oriental region. Moreover, our analysis indicated that the distributional changes in the Pierinae lineages were correlated with their host plants, the Brassicales, which underwent rapid and dramatic diversification in the late Cretaceous, especially in the K–Pg boundary, as shown in a previous study [67]. For example, in the tribes Anthocharidini and Pierini, the MRCA of three clades (*Eroessa* + *Hesperocharis*), ((*Melete* + (*Pereute* + *Leodonta*) + ((*Neophasia* + *Eucheira*) + (*Charonias* + (*Catasticta* + *Archonias*))))), and (*Ascia* + (*Ganyra* + (*Hypsochila* + ((*Theochila* + *Tatochila*) + (*Phulia* + (*Infraphulia* + (*Piercolias* + *Pierphulia*))))))) dispersed from the Oriental region into the Neotropical region at about 34 Mya, shortly after the Brassicales extended their distribution to South America [67]. This probably occurred through the BLB or stepping stones between these two zoogeographical regions [70,72,73], in contrast to the use of the Thulean Land Bridge (TLB) [74,75,76]. However, the MRCA of some lineages, such as the *Euchloe*, *Anthocharis*, *Zegris*, *Aporia*, *Pontia*, and *Reliquia*, returned to the Palearctic region from the Oriental region between 27.5 and 16.9 Mya during the Oligocene to Miocene eras, which was also correlated with the dispersal of their host plants Brassicales to Europe by the closure of the Turgai Straits during the Oligocene era [63]. Soon after, the subfamily Pierinae began to rapidly diversify into their extant lineages of current distributions (Figure 7b).

## 4. Conclusions

In this study, for the first time, we obtained the complete or nearly complete mitochondrial genomes of 100 pierid butterfly species, covering three subfamilies and fifty-six genera. Based on this relatively larger-scale dataset, we conducted comparative mitogenomic, phylogenetic, and phylogeographic analyses to further clarify the spatio-temporal evolutionary patterns of the Pieridae. Our results showed that the GC content of PCGs in Pierinae was elevated compared with that of the Coliainae, which might be correlated with their relatively higher mutation rate. All the obtained Pieridae phylogenetic trees in our study were identical in topology across the different methods (ML and BI phylogenetic analyses) and datasets (13_PCGs and 15_genes), providing a phylogenetic backbone of Pieridae in terms of mitogenomics. However, some minor incongruencies with previous studies were observed. Our phylogenetic analysis showed that the spatio-temporal evolutionary patterns of Pierinae were closely associated with the evolution of their host plants, the Brassicales, as well as the corresponding geological events and environmental changes over time and in different locations throughout Earth’s history.

## Data Availability

The complete mitochondrial genomes newly sequenced in this work have been deposited in GenBank under the accession number ranging from OP779721 to OP779726.

## Supplementary Materials

The following supporting information can be downloaded at: https://www.mdpi.com/article/10.3390/genes14010072/s1, Table S1: Details of species and mitogenomes of Pieridae and outgroups used in this study. Table S2: Partition schemes and best-fitting models for phylogenetic analyses. Figure S1: Saturation plots for the 13_PCGs, 15_genes, and 13 PCGs_codon3 performed in DAMBE 7 with the GTR model selected as a reference model. S: transition rate; V: transversion rate. Figure S2: Phylogenetic relationships of the 100 Pieridae species from Bayesian inference (BI) analysis based on 13_PCGs; posterior probabilities are indicated at each node. Figure S3: Phylogenetic relationships of the 100 Pieridae species from maximum likelihood (ML) analysis based on 15_genes; bootstrap values are indicated at each node. Figure S4: Phylogenetic relationships of the 100 Pieridae species from Bayesian inference (BI) analysis based on 15_genes; posterior probabilities are indicated at each node.
