# Supplementary material for: Spatio-Temporal Evolutionary Patterns of the Pieridae Butterflies (Lepidoptera: Papilionoidea) Inferred from Mitogenomic Data
Source: Genes (Basel). 2022 Dec 26;14(1):72. doi: 10.3390/genes14010072 (PMC9858963; doi:10.3390/genes14010072)
Supplement: Supplementary file 1 [file genes-14-00072-s001.zip › Supplementary_Figure.pdf]

Supplementary Materials

Spatio-Temporal Evolutionary Patterns of the Pieridae Butterflies (Lepidoptera: Papilionoidea) Inferred from Mitogenomic Data

Supplementary Table S1. Details of species and mitogenomes of Pieridae and outgroups used in this study.

Supplementary Table S2. Partition schemes and best-fitting models for phylogenetic analyses.

Supplementary Figure S1. Saturation plots for the 13_PCGs, 15_genes and 13 PCGs_codon3 performed in DAMBE 7 with the GTR model selected as a reference model. S: transition rate; V: transversion rate.

Supplementary Figure S2. Phylogenetic relationships of the 100 Pieridae species from Bayesian inference (BI) analysis based on 13_PCGs, posterior probabilities are indicated at each node.

Supplementary Figure S3. Phylogenetic relationships of the 100 Pieridae species from Maximum Likelihood (ML) analysis based on 15_genes, bootstrap values are indicated at each node.

Supplementary Figure S4. Phylogenetic relationships of the 100 Pieridae species from Bayesian inference (BI) analysis based on 15_genes, posterior probabilities are indicated at each node.

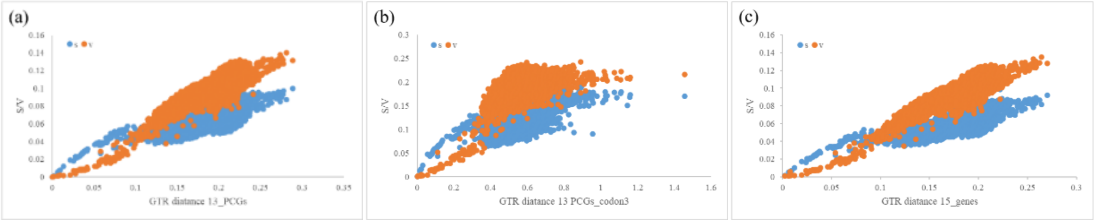

Figure S1. Saturation plots for the 13_PCGs, 15_genes and 13 PCGs_codon3 performed in DAMBE 7 with the GTR model selected as a reference model. S: transition rate; V: transversion rate.

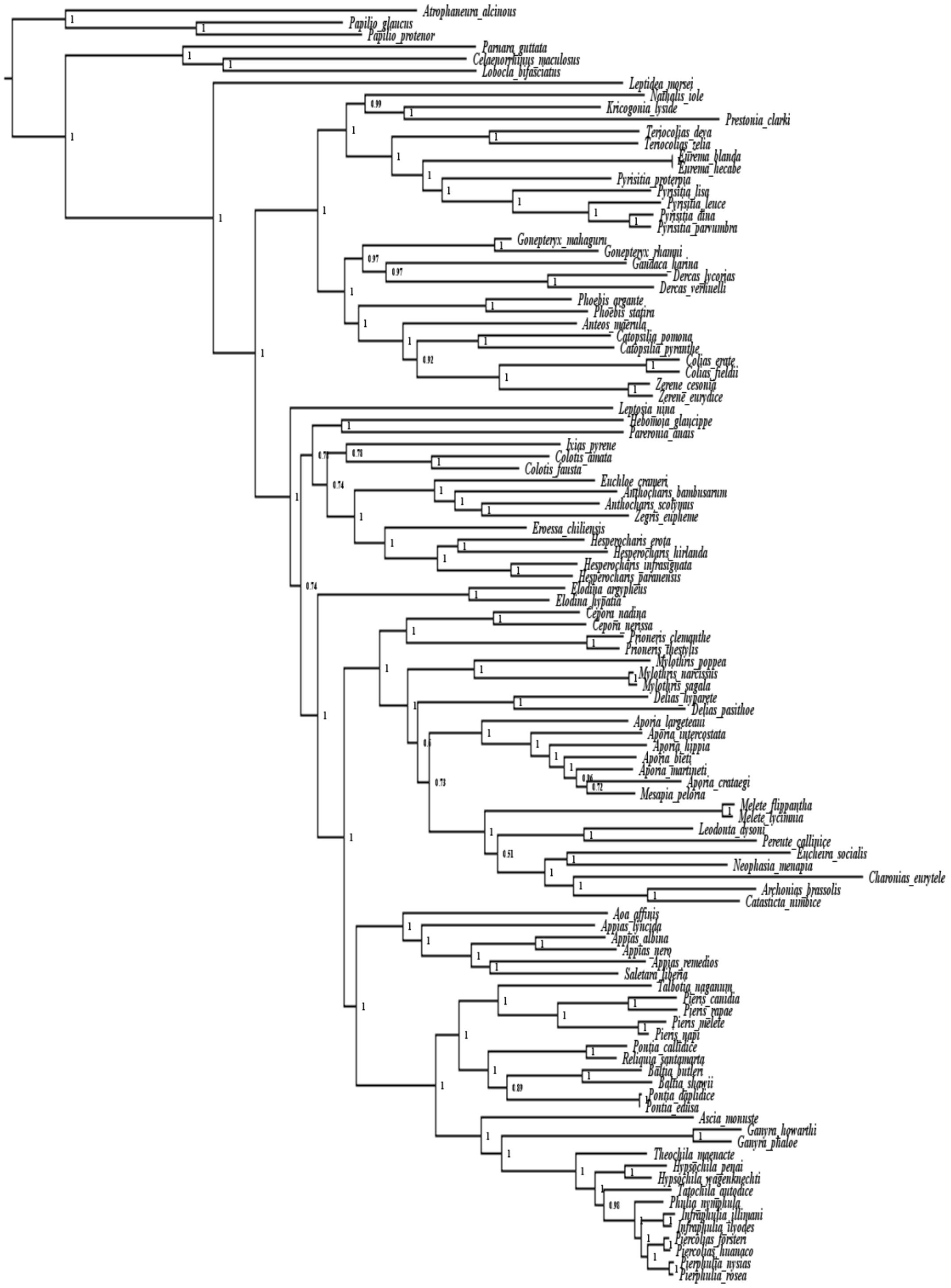

Figure S2. Phylogenetic relationships of the 100 Pieridae species from Bayesian inference (BI) analysis based on 13 mitochondrial protein coding genes, posterior probabilities are indicated at each node.

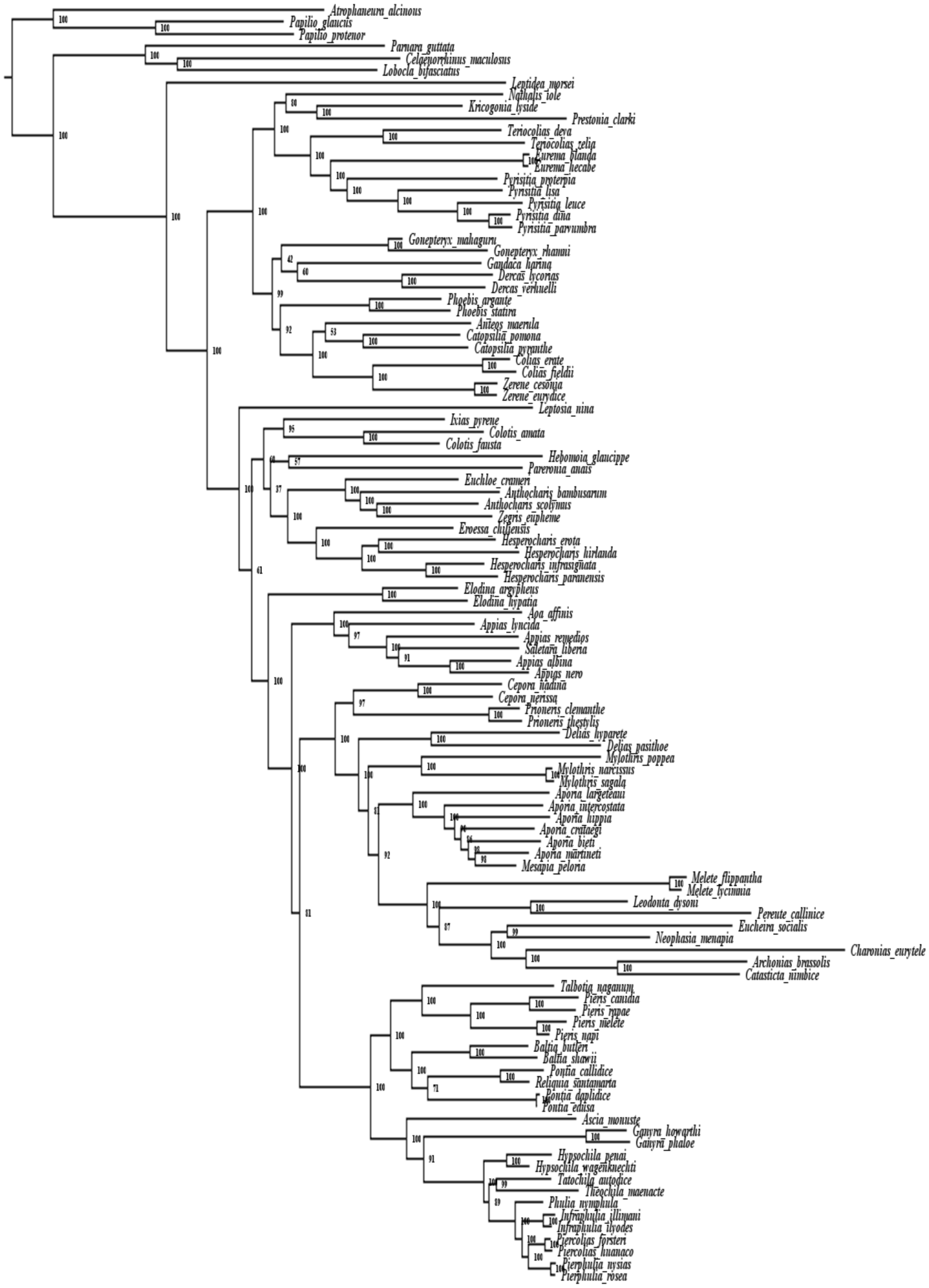

Figure S3. Phylogenetic relationships of the 100 Pieridae species from Maximum Likelihood (ML) analysis based on 15 genes (13 mitochondrial protein coding genes and two rRNAs), bootstrap values are indicated at each node.

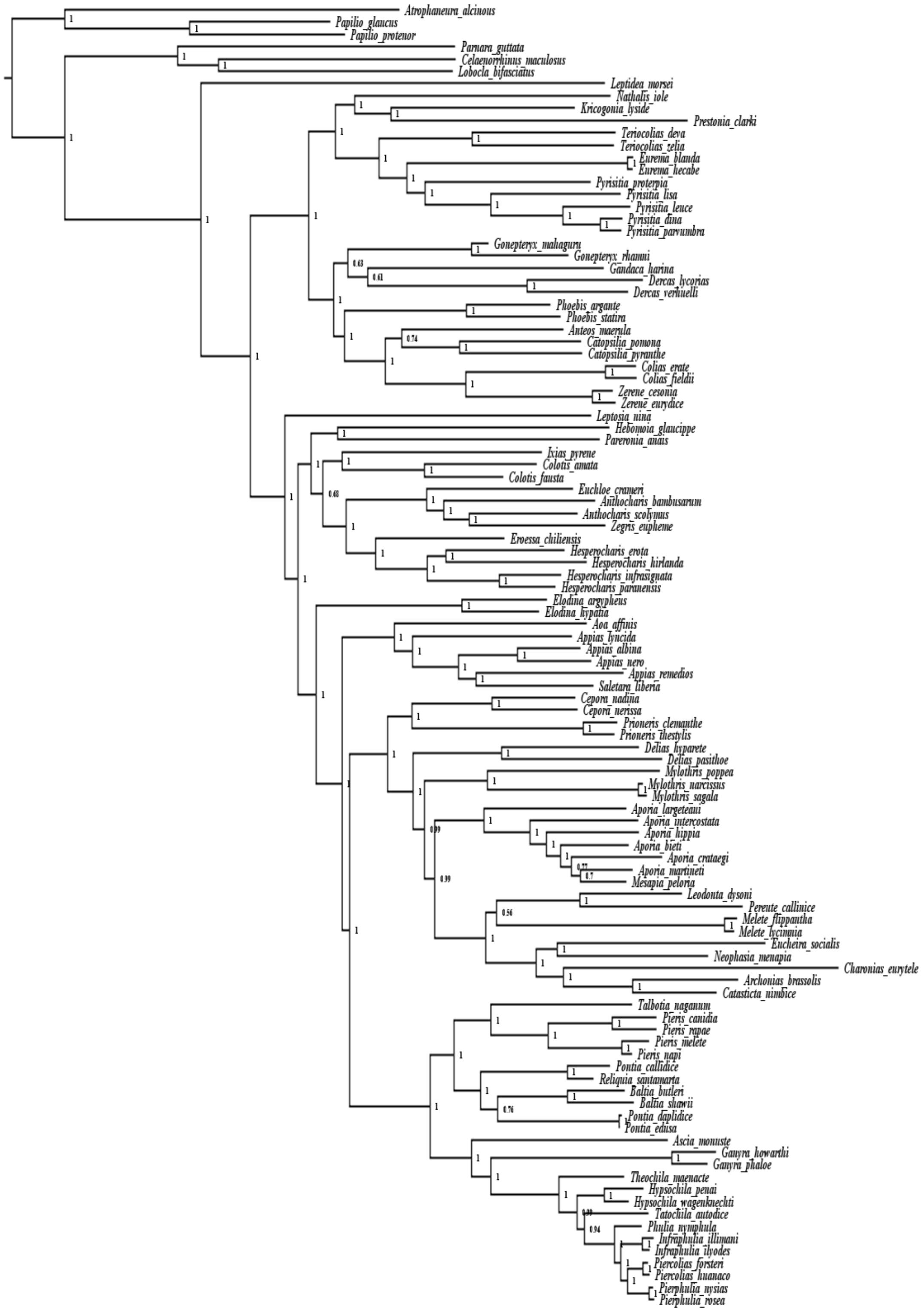

Figure S4. Phylogenetic relationships of the 100 Pieridae species from Bayesian inference (BI) analysis based on 15genes (13 mitochondrial protein coding genes and two rRNAs), posterior probabilities are indicated at each node.
